# New healthcare payment models: risk scores aren’t enough to guide resource allocation

**DOI:** 10.1038/s41598-025-04285-w

**Published:** 2025-05-29

**Authors:** Christopher Crowley, Bradley Harner, Amy R. Stuck, Tyler Kent

**Affiliations:** West Health Institute, La Jolla, San Diego, CA USA

**Keywords:** Healthcare economics, Resource allocation, Older adults, Hospital utilization, Risk, Health care economics, Health policy

## Abstract

Around the world, aging populations compel healthcare delivery systems to improve how they allocate increasingly scarce resources. In parallel, economic pressures motivate healthcare payors and policy makers to adopt global budgeting and accountable payment models based on actuarial risk. We investigated whether these risk-based approaches could apply to healthcare resource allocation. Because a significant portion of healthcare resources for older adults is currently associated with potentially avoidable hospital admissions, we focused our investigation on allocating care coordination resources targeted toward those most likely to be admitted. Using a computational risk-based analysis of claims data, we found the 20% highest expected hospital utilization segment had an average hospitalization rate of over 68% per year, compared to 27% for the overall study population. However, only half of all hospitalizations in the study population were accounted for in the top 20% risk segment. Additionally, 63% of beneficiaries in the top 20% risk segment experienced zero hospitalizations. Our results indicate that risk-based resource allocation may fail to target some high hospital utilizers while allocating resources to many who are never hospitalized. These results further indicate that risk-based expectation of hospital utilization may be insufficient as a basis for effective allocation of care coordination resources.

## Introduction

Around the world, populations are aging, resulting in a renewed focus on the economics of healthcare delivery. As the global population of older adults is projected to grow to 1.4 billion by 2030^1^, there is a particular need for research directed toward improved resource allocation. A significant portion of healthcare resources for older adults is currently associated with hospitalizations, due in part to a higher prevalence of advanced chronic disease, often compounded by demographic and social risk factors. In the United States, for example, adults aged 65 and over account for more than 42% of all hospital utilization^2^, despite representing less than 20% of the population^3^. Hospital-based care for older adults is costly, with 47% of hospital costs in the United States being borne by Medicare^4^, the national insurance program that includes adults aged 65 and over and is administered by the Centers for Medicare and Medicaid Services (CMS). Importantly, although hospital-based care may be needed in many cases, it is not necessarily the best option for managing frailty, chronic disease exacerbations, and social determinants of health. For older adults, hospitalizations are specifically associated with adverse outcomes including delirium, hospital-acquired infections, and falls^5^. Due to both the high cost and associated risk factors, there is a need to develop lower-cost options that more efficiently allocate medical resources and reduce reliance on hospital-based care for older, more complex populations. The objective for the present investigation is to reduce the overuse of hospital-based care and improve patient experience.

Hospitals have demonstrated an ability to extend their sites of care to include the homes and communities where older adults live^6,7^. However, for some of the highest-risk older patient populations, evidence suggests that hospital-managed care may be avoided altogether through appropriately allocated home, community and ambulatory care resources^8,9^. In considering such evidence, it is important to note that improving efficiency of care delivery requires providers both having agency over when and for whom to allocate community-based resources, as well as concomitant incentives to reduce the overall cost to care for a population. In the Programs of All-Inclusive Care for the Elderly (PACE)^10^, for example, Interdisciplinary Teams (IDT) operate in conjunction with fully capitated risk-based payments covering the total-cost-of-care (TCOC) for all participating members. Beyond PACE, risk-based payment models are expanding to include global budgets^11^ and Accountable Care Organizations (ACOs), now adopted widely in the U.S. and under consideration in other countries^12^. In the U.S., CMS has set a goal that every beneficiary in traditional Medicare receive coordinated care through providers in accountable care relationships by 2030^13^.

Whenever providers assume financial responsibility related to TCOC, risk adjustment is necessary to predict expected spending resulting from factors such as advanced age, socioeconomic burdens, and prevalence of chronic disease^14^. In the U.S., the CMS hierarchical condition category (CMS-HCC) risk adjustment model is used to prospectively determine capitated payment levels in PACE as well as benchmarks for financial performance in ACOs. The CMS-HCC specifically uses demographic and diagnostic data obtained from a base year to determine expected spending in a subsequent performance year^15^. The specific mappings from base year to performance year are determined through historical calibration data used to train standard linear regression and other related actuarial models^16^.

Importantly, risk adjustment is a financial tool, designed to ensure fairness in payment across populations. It is calibrated to expected cost versus future need, individual actionability or patient-level decision-making. Yet in many real-world contexts, this distinction is blurred. Risk scores—especially when viewed at the individual level—are often interpreted by providers as indicating a need for proportionate care resource allocation. Even if they don’t literally use risk scores, many providers adopt related prospective criteria that conflate risk and resources. In addition, certain policies may tie billable services to generalized risk-related parameters such as the 2015 introduction of Chronic Care Management (CCM) by CMS. Under CCM, providers can bill an additional $60-$100 per patient per month for managing patients with multiple chronic conditions^17^. While intended to promote proactive care, the practical effect has often been to incentivize uniform, revenue-capturing strategies that may be misaligned with the intrinsically heterogeneous nature of patient-centered care delivery. As a result, as one analyst has observed, care coordination programs often fail to reduce total spending—not because they lack clinical benefit, but primarily because the number-needed-to-treat (NNT) is high and the intervention itself incurs real costs, limiting the financial return even when patient outcomes improve^18^.

To compound matters, standardized interventions have become entangled with the logic of risk score intensification. A recent investigation highlighted how nurse home visits in community settings are increasingly used not to coordinate care, but to generate risk markers and diagnoses not typically represented in the original CMS-HCC calibration cohort^19^. This paper recognizes that challenges surrounding coding intensity and model calibration must be addressed. But resolving those issues is not the same as making risk scores clinically actionable. The more fundamental distinction is that CMS-HCCs and related prospective determinants of risk are not care signals.

Our investigation is intended to get at the heart of this matter quantitatively. Using a generalized risk-based methodology, we examine what occurs when actuarial risk scores are treated as if they could guide care delivery. By deliberately using one model for the purpose of another, we aim to illustrate that prospectively determined risk is not enough to guide resource allocation. Specifically, and as elaborated below, we assess whether progressively higher cumulative risk strata—constructed using standard regression models—correspond to consistently actionable differences in hospital utilization. Thus, our investigation could shed light on previously observed challenges of cost-effectiveness as identified in^18^. In addition, our investigation could inform additional factors needed to target care coordination and related resources in ways that more cost-effectively reduce hospital utilization. To provide real-world context, our narrative implicitly applies to allocating care coordination and related clinical resources. In the discussion section, we broaden this framing to include a wider range of interventions, including those that might be categorized as preventive strategies such as screening, risk-based outreach, or early engagement efforts, all of which may be effectively deployed upstream of a more imminent adverse event like hospitalization.

## Methods

We designed a computational economic study using a retrospective observational cohort of Medicare beneficiaries in the U.S. We specifically used a risk-based methodology in which the CMS-HCCs were defined as independent variables spanning a well-established range of demographic and diagnostic groupings from a base year. Our dependent variables were hospital utilizations in a subsequent performance year. Thus, while the CMS-HCC variables are traditionally used to obtain expected cost in a performance year, we directly applied an actuarial regression model to obtain expected performance year hospital utilization. We then compared expected versus actual observed hospital utilization in our study cohort. Using expected hospital utilization, we defined five quintiles representing segments of expected hospital utilization ranging from highest to lowest. We evaluated the performance of our approach using the positive predictive values and R^2^, which are the same statistical measures used to characterize the CMS-HCC model in its traditional determination of expected costs^15^. In addition to calculating expected and observed hospitalizations, we developed cumulative risk segments, similar to those used in^20,21^. We then inferred efficiency of using our cumulative risk segments as a basis for allocating care coordination and related resources by considering the following outcomes:

- How many of the hospitalizations experienced by 100% of the population end up being included in the highest risk segment
- How many beneficiaries within the highest risk segment actually experience zero hospitalizations

Western IRB Copernicus Group (WCG) served as the Institutional Review Board (IRB) for this research. The WCG IRB waived informed consent for participation and determined that our investigation was exempt under 45 CFR § 46.104(d)(4) of the US Code of Federal Regulations because the identity of the human subjects could not be ascertained or re-identified.

### Setting

**Fig. 1:**
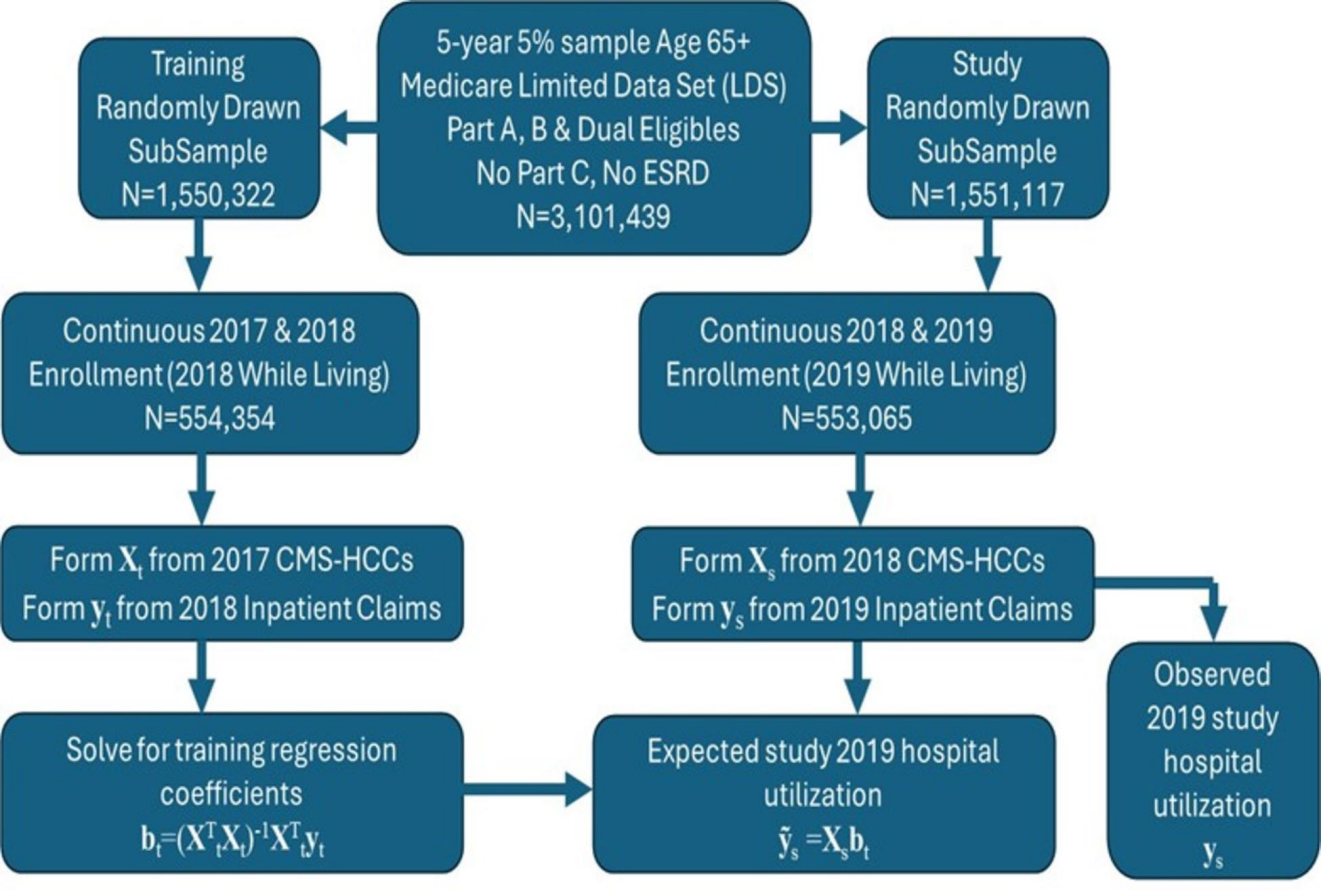
Data and statistical analysis methodology using the Medicare Limited Data Set. Claims data were stored in a secure cloud-based relational database (Snowflake version 8.41.2). Linear regression was carried out in Jupyter notebook version 6.4.12 running Python version 3.9.7 with training data from 2017 and 2018 generating expected 2019 hospital utilization for comparison with observed 2019 hospital utilization.

Our investigation was based on an initial 5-year national limited data set comprising a standardized 5% sample of all Medicare FFS (fee-for-service) claims and demographic data, curated by RESDAC and validated as being representative of the 100% population^22^. We established data and analysis pathways for our investigation as indicated in Fig. 1 and summarized as follows. All limited data set sample claims data were housed in a cloud-based relational database and included institutional and clinician-based services. The limited data set included diagnostic information necessary to determine the CMS-HCC groupings to be used as independent variables from a base year. The initial data sample also included hospital utilization, used as the dependent variable in a subsequent performance year. The initial sample was limited to Medicare beneficiaries aged 65 and over who exhibited enrollment in Medicare Parts A and B anywhere in the years spanning 2017–2019. We included beneficiaries with state buy-in indicators representing dual-eligible beneficiaries qualified for both Medicare and Medicaid services. We excluded beneficiaries with any enrollment in Medicare Advantage (Part C) because we did not have full access to their claims data. We also excluded beneficiaries with end-stage renal disease because CMS uses a distinct HCC model for these beneficiaries.

We followed the same calibration steps used in standard actuarial practice^15^. Specifically, we split the initial sample population into a training population and a study population, using a random number generator from the relational database to assign an even or odd random integer to every beneficiary in the sample population. As a result of the randomization, there was no overlap between the training population and the study population. However, due to their originating from the same initial 5% sample, the training and study populations shared the same representative demographic, enrollment, and diagnostic characteristics.

Following randomization, we imposed additional constraints to further refine the definitions of the training and study populations. We first refined the training population by including only those continuously enrolled in Medicare Part A and B over the years 2017 and 2018. We also included beneficiaries who passed away in 2018, provided they were continuously enrolled in Medicare Parts A and B while living. We designated 2017 to be a base year and limited the training set data, drawing independent variables only from the base year claims. We then designated 2018 to be a performance year, further limiting our training set data by obtaining our dependent variables (i.e., the expected hospital utilization) only from these subsequent performance year inpatient claims. For the study population, we imposed similar limitations, but we used 2018 as the base year independent variables while the performance year dependent variables (hospitalizations) were drawn from 2019 inpatient claims. Thus, for study data, we avoided using the same base year/performance year pairings as those used in the training set.

### Analysis

For every member of the training population, we obtained 2017 base year Medicare demographic and ICD-10 diagnosis codes from the limited data set Inpatient, Outpatient, and Carrier claims. We converted these data elements into HCCs using the published model mappings for CMS-HCC version 22 from the Centers for Medicare and Medicaid Services^15^. We obtained a mapping of expected 2018 hospital utilization as a function of the 2017 CMS-HCCs using a linear regression statistical model as described in^16^ and summarized as follows.

First, we formed a training matrix **X**_t_, having one row for every member of the training population with columns representing the 2017 base year HCCs corresponding to that member. We also formed a training column vector **y**_t_, also having one corresponding row for every member of the training set, with values representing the count of 2018 performance year hospitalizations for that member. To apply the linear regression statistical method, we formed a matrix equation representing the mapping from base year independent variables to performance year dependent variables:

1 $${\mathbf{X}}_{{\text{t}}} {\mathbf{b}}_{{\text{t}}} \approx {\mathbf{y}}_{{\text{t}}}$$

In Eq. (1), **b**_t_ is a column vector representing the regression coefficients. We note that there are typically many more members in the training population than HCCs, so Eq. (1) represents a heavily overdetermined system and may not have an exact solution.

As described in^16^ we obtained the least squares solution to Eq. (1), which is represented by

2 $${\mathbf{b}}_{{\text{t}}} = \left( {{\mathbf{X}}^{{\text{T}}}_{{\text{t}}} {\mathbf{X}}_{{\text{t}}} } \right)^{{ - {1}}} {\mathbf{X}}^{{\text{T}}}_{{\text{t}}} {\mathbf{y}}_{{\text{t}}}$$

where **X**_t_^T^ is the transpose of the matrix **X**_t_.

We next obtained a corresponding matrix **X**_s_ from the study set data, using the 2018 CMS-HCCs. We also obtained the study set column vector **y**_s_ representing the actual observed 2019 hospitalizations. We then used the regression coefficients **b**_t_ from the training set to obtain the expected 2019 hospital utilization in the study set:

3 $$\bf \tilde{y}_{{\text{s}}} = {\mathbf{X}}_{{\text{s}}} {\mathbf{b}}_{{\text{t}}}$$

In Eq. 3, the tilde symbol **ỹ**_s_ is used to distinguish the expected 2019 performance year hospital utilization from **y**_s_, representing the actual observed 2019 hospitalizations. To evaluate how the expected hospital utilization compares with the actual, we used statistical methods similar to those employed by CMS to evaluate the CMS-HCC model^15^. Specifically, we divided the population into 5 quintiles, based on the expected values ordered from lowest to highest. We then plotted the average observed and average expected utilization within each of the quintiles. We then calculated the predictive ratios, representing the ratio of average expected to average observed values in each of the quintiles. We also provided a scatter plot of the individual observed hospital utilizations within each quintile to illustrate the differences between individual and quintile averages. Corresponding to the scatter plot, we calculated error bars and the R^2^ statistic as used in^15^ to provide an overall indication of how well the expected utilization **ỹ**_s_ matched the actual utilization **y**_s_ at an individual level. Finally, we obtained five progressively narrower cumulative risk segments consisting of cumulative quintiles of the 100% as well as the top 80%, 60%, 40%, and 20% expected utilizers of hospital-based care. We plotted histograms of actual observed hospital utilization in each of these cumulative risk segments. The use of cumulative risk segments allows us to more directly compare the highest-risk segment with the overall 100% cumulative segment used in^20,21^. Finally, we also calculated the percentages of hospitalizations experienced by the population that end up being included in the cumulative risk segments as well as how many beneficiaries within the cumulative risk segments actually experience zero hospitalizations.

## Results

**Fig. 2:**
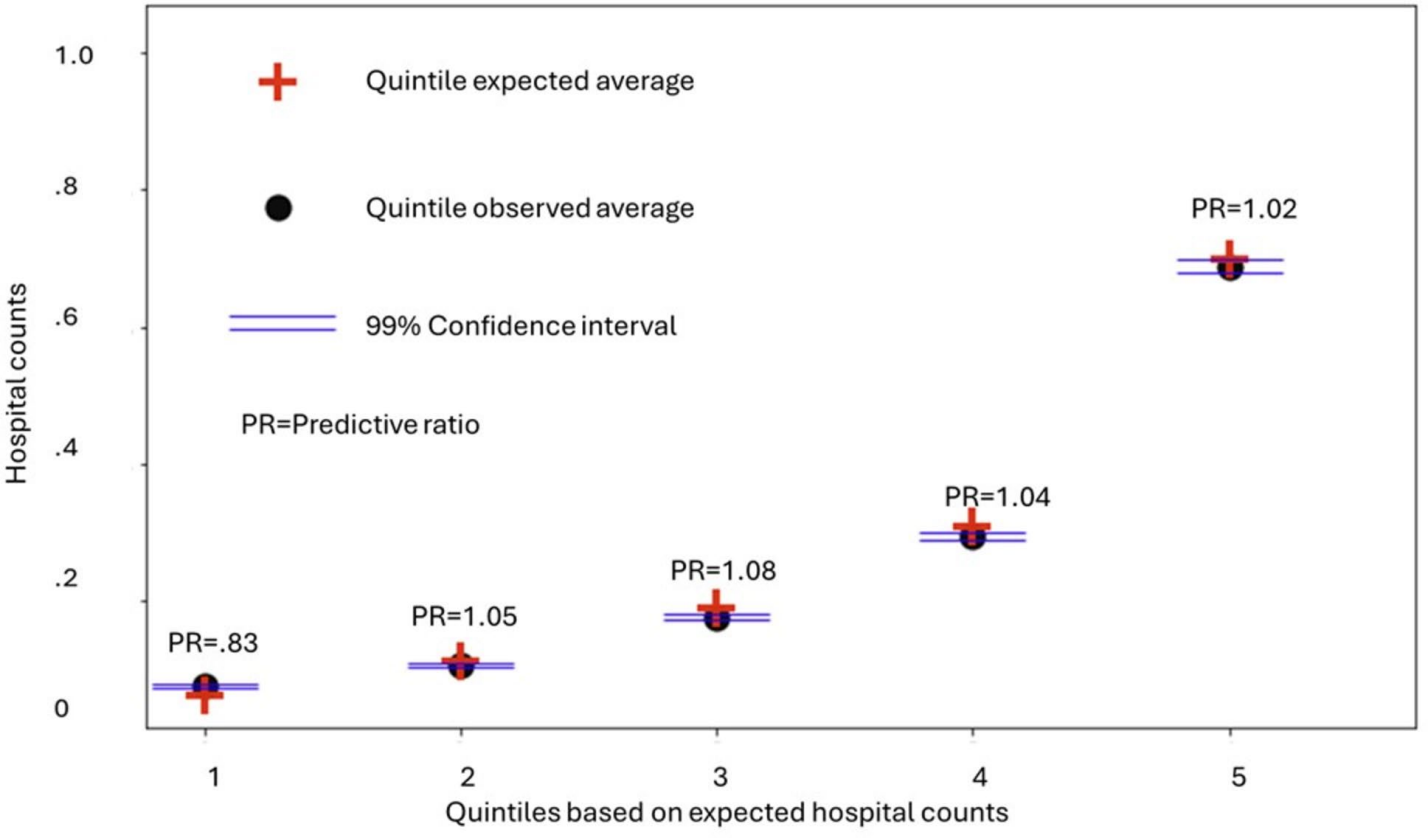
Expected and observed quintile averages for 2019 hospital utilization counts, sorted by the five quintiles of expected utilization. For each quintile, predictive ratios are indicated along with confidence intervals representing the uncertainty about the average of the observed samples. Data visualization created by CC, BH and TK using Matplotlib version 3.5.1 in Python.

**Fig. 3:**
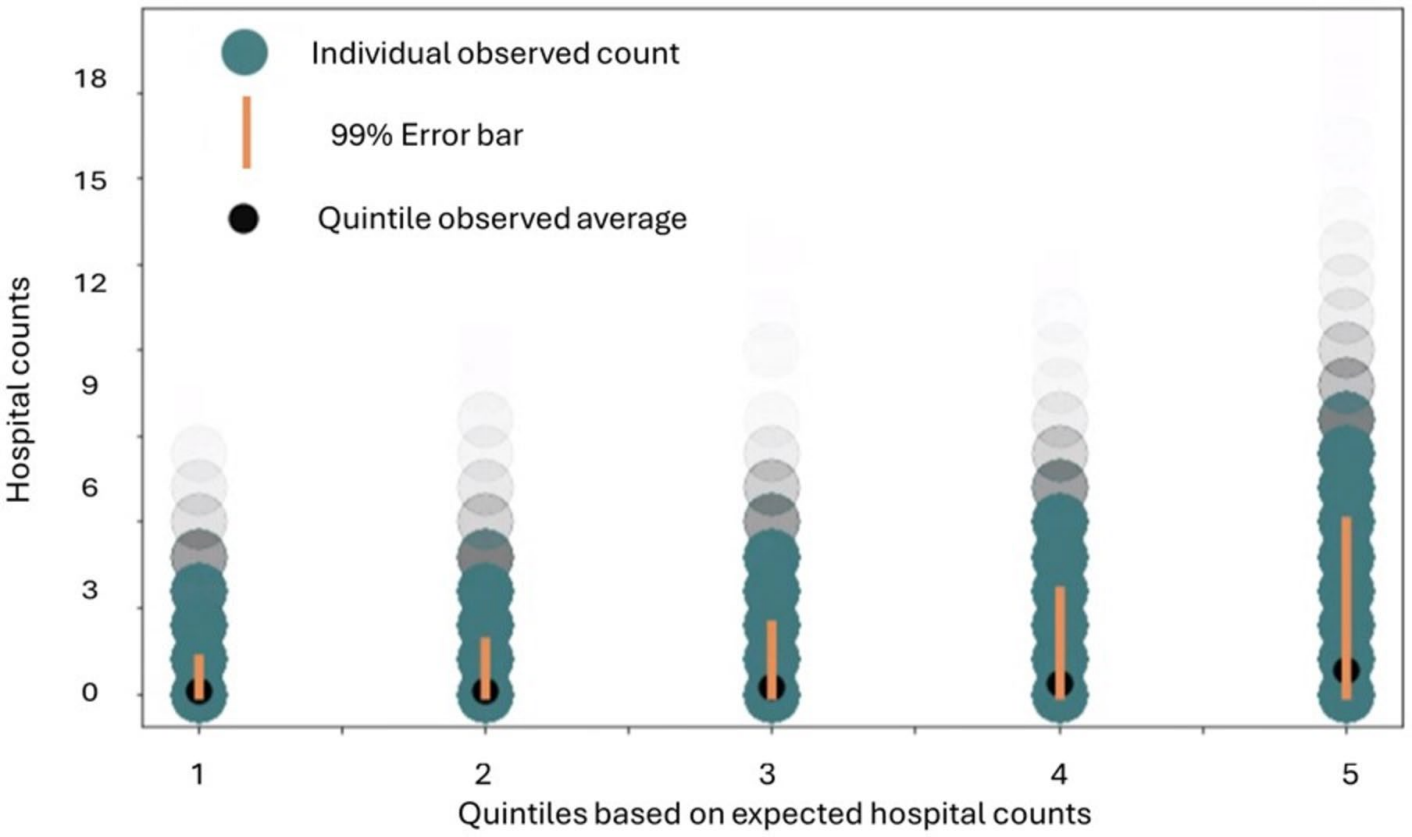
Actual observed individual counts of hospital utilization using a scatter plot format sorted by the five quintiles of expected utilization. The intensity of each plotted data point represents the number of individuals exhibiting the corresponding count of hospitalizations. For each of the quintiles, error bars indicate the boundaries containing 99% of the individual hospital count outcomes. Average observed values shown from Fig. 2 are also plotted in Fig. 3. Data visualization created by CC, BH and TK using Matplotlib version 3.5.1 in Python.

Figure 2 shows the expected and observed quintile averages for 2019 hospital utilization counts, sorted by the five quintiles of expected utilization. For each quintile, predictive ratios are indicated along with confidence intervals representing the uncertainty about the average of the observed samples. Figure 3 shows the actual observed individual counts of hospital utilization using a scatter plot format again sorted by the five quintiles of expected utilization. For each of the quintiles, error bars indicate the boundaries containing 99% of the individual hospital count outcomes. Average observed values shown from Fig. 2 are also plotted in Fig. 3. As indicated by both the long error bars and the extensive distribution of individual hospital counts in Fig. 3, there is a significant amount of individual variation despite the similarities between expected and actual group level averages for each of the quintiles indicated in Fig. 2. Figure 3 also illustrates the extensive asymmetry in the individual observed hospital counts. Specifically, in several of the quintiles, and especially in the top 20% highest quintile, there are small numbers of individual patients that exhibit actual observed hospitalization counts more than 10 times higher than the expected average for the quintiles—a result consistent with characterizations of risk indicated in^15^. As an overall statistical measure of the degree to which individuals plotted in Fig. 3 exhibit actual hospital utilization that differs from the average, we obtained R^2^ = 0.12, which is similar to the R^2^ statistical measures reported in^15^.

**Fig. 4:**
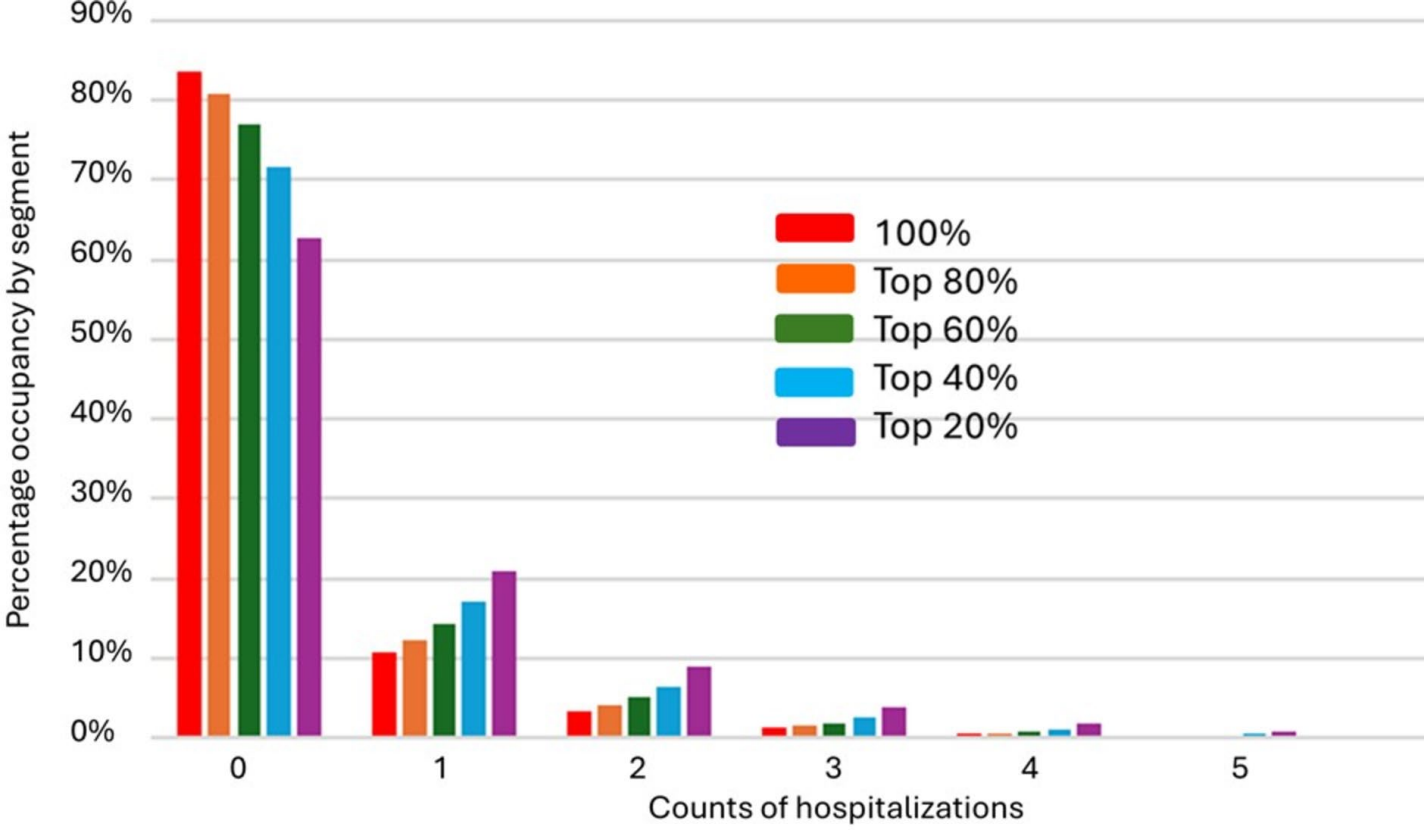
Distributions of actual individual hospital utilization in a histogram plot format, grouped by the cumulative risk segments. Data visualization crated by CC using Microsoft® Excel® for Microsoft 365 MSO (Version 2409).

We next used the five quintiles to compute the cumulative risk segments. The highest 20% cumulative risk segment directly corresponded to the highest 20% quintile, while the second highest cumulative risk segment represented the combined the highest and second highest quintiles, and so forth. The lowest cumulative risk segment comprised of 100% of the study sample. Figure 4 shows percentage distributions of actual individual hospital utilization in a histogram plot format, grouped by the cumulative risk segments. Table 1 provides a comparison between the expected and observed rates of hospitalization across all of the cumulative risk segments, ranging from the total study population (100%) up to the top 20%.

**Table 1:** Numbers of beneficiaries, expected and actual hospital counts and percentages of total hospitalizations contained in each of the respective cumulative risk segments.

	100%	Top 80%	Top 60%	Top 40%	Top 20%
Number of Beneficiaries	553,065	442,452	331,839	221,226	110,613
Expected Number Hospitalizations	151,793	144,824	132,455	111,425	77,386
Expected Hospitalization Rate	27.4%	32.7%	39.9%	50.4%	70.0%
Actual Number Hospitalizations	148,454	140,082	128,322	108,833	76,171
Actual Hospitalization Rate	26.8%	31.7%	38.7%	49.2%	68.9%

Table created by CC using Microsoft® Excel® for Microsoft 365 MSO (Version 2409).

Figure 4 indicates the degree to which individual members within each of the risk segments exhibited highly skewed, heterogeneous utilization. Considering the 20% highest expected utilization risk segment, Table 1 and Fig. 4 indicate that the average hospitalization rate is about 69% per year. The other segments exhibit lower hospitalization rates, with the average for the 100% study population being 27%. Thus, the top 20% risk segment has a more than two times higher probability of being admitted to the hospital compared with the 100% cumulative risk segment. However, if we consider our first outcome—how many of the hospitalizations experienced by the total population (148,454) are experienced by a given risk segment—Table 1 indicates that only about half (76,171) end up being included in the top 20% risk segment. Additionally, if we consider our second outcome—how many beneficiaries within a given segment actually experience zero hospitalizations—Fig. 4 indicates that about 63% of the top 20% risk segment never go to the hospital. Thus, despite taking into account a broader range of risk factors, discrepancies between expected and actual hospitalizations appear to cause the risk-based segmentation to both exclude beneficiaries who do go to the hospital and provide resources to many who are never hospitalized.

## Discussion

Our results confirm that while actuarial risk scores perform well in aggregate, they do not reliably identify which individuals will experience costly hospital-based events. While our study used the CMS-HCC model to illustrate the point, our results may be representative of all prospective approaches to allocating care coordination and related resources. This distinction lies at the heart of our investigation and motivates the central argument of this paper: risk scores aren’t enough to guide resource allocation. Our findings suggest a need for more adaptive, system-level strategies that respond to endogenous information, meaning clinical or behavioral signals that emerge after group-level risks have been determined. Importantly, embedding endogenous information in payment models is not advisable due to a resulting incentive to grow utilization. As a result, there is a lack of specific resource guidance intrinsic to risk-based payment models. But once risk adjustment determines fair-price contract pricing, global budgets or benchmarks, risk-bearing providers are uniquely positioned to observe these endogenous signals in real time and act on them accordingly. By designing systems that track needs and deploy resources dynamically, value-based organizations can fulfill their potential: not just bearing risk, but actively managing it at a system level.

Examples of system-level care design that responds to endogenous events are represented in the literature. For example, geriatric emergency departments (GEDs) provide senior-appropriate care in which traditional ED resources are augmented with geriatric nurses, care managers, and social workers. These resources are activated upon arrival to the GED, as opposed to being assigned based on risk alone. A recent peer-reviewed study of a Level 1 geriatric emergency department (GED) found that a comprehensive geriatric assessment (CGA) was associated with a 13% absolute reduction in hospital admission risk and approximately $1,900 per patient in Medicare cost savings^23^. Notably, the resources deployed in the GED are comparable, albeit not necessarily identical to those employed in traditional Chronic Care Management (CCM) and related programs. What distinguishes the GED-based models is not the personnel themselves, but the timing and rationale for their deployment. It is also notable that both the mechanisms and counterfactual-based tabulation of realized savings may be clearer when tied to event-triggered resource allocation approaches.

Notwithstanding evidence in support of system-level approaches, it is important to acknowledge that such approaches may not be widely adopted. One reason may be that intercepting patient needs as they emerge requires more comprehensive system changes than simply assigning care management resources based on prospective risk. For example, for every patient intercepted in a GED, a range of alternative disposition options may be needed, with streamlined referral pathways and, in some cases, specific order sets. Building this infrastructure is more challenging than adding independent care coordination service lines. Moreover, without widespread implementation of system-level changes in care delivery, ACOs have already garnered over $11 billion in cumulative shared savings payments since program inception^24^. As a result, incentives to change the status quo may be limited. Notwithstanding, analysts have raised important concerns about the basis of these reported savings, noting for example concerns about a lack of counterfactuals^25^. Finally, the limited adoption of system-level care redesign may also reflect persistent conflation of prospective risk and clinical resource allocation—the conceptual distinction that our investigation seeks to illuminate.

## Limitations

Our data and analysis had several limitations. A primary limitation is that we evaluated only the effectiveness of risk-based enrollment criteria—we did not actually evaluate the effects of allocating care coordination and other resources directed toward reducing hospital admissions. Moreover, in some of our data, care coordination and other resources may have already been deployed, causing over-estimation of the numbers of beneficiaries who would experience zero hospitalizations in the absence of exposure to such resources. Additionally, at the time of our investigation, our data were almost 4 years old, which may be somewhat out of date, albeit potentially representing pre-COVID-19 pandemic patterns that may be relevant to the present day. Finally, we drew our inferences about prospective approaches to resource allocation using only one representative example obtained from the CMS-HCC model. Additional risk-based groupings are available, either in addition to or different from the CMS-HCC, potentially with different outcomes. Similarly, while we used a linear regression methodology, other standard actuarial approaches such as generalized linear regression^16^ could be considered.

Our inferences concerning system-based approaches to resource allocation also have significant limitations. In particular, it is important to acknowledge that relying solely on late-stage signals would yield high predictive accuracy but fewer opportunities for proactive intervention, potentially leaving hospitalization and other default care pathways as the only available responses. Our findings should not be interpreted as support for waiting until significant care needs fully declare themselves.

Another limitation is that our focus was implicitly on care coordination and other related resources. In other words, our findings support the development of adaptive allocation of care coordination resources in a manner that balances the number-needed-to-treat (NNT), cost to treat, and net impact toward objectives. Our findings should not be construed as discounting the inherent tradeoffs between early inclusion, predictive precision, and the goal of effective preventive action, especially outside of the realm of care coordination. Vaccines, for example, are cost-effective when allocated broadly even when only a few of the recipients are actually exposed to the target pathogen. Broadening the implied scope of resources beyond specific clinal care coordination resources, our study would in no way preclude low cost proactive, upstream strategies rather than relying solely on event-triggered interventions (e.g., those initiated only after GED visit). For example, our study would indicate using community-based, non-clinical surveillance and early outreach when available to help identify at-risk individuals before they escalate to critical events. While such an approach might include numerous individuals who do not progress to adverse outcomes, the resulting trade-off may be intentional, acceptable, and, in many cases, cost-effective.

## Conclusions

Our investigation examined whether actuarial risk scores—specifically those from the CMS-HCC model—can serve as a sufficient basis for allocating hospital-avoidance resources. We found that, while the model performs well in the aggregate, individual-level utilization within risk segments is highly heterogeneous. In every risk stratum, a majority of patients had no hospitalizations, while a small minority accounted for most of the observed events. This confirms that prospective risk scores—however well calibrated—cannot reliably identify which individuals will benefit from targeted interventions. These findings reinforce a critical distinction: prospective risk can support payment fairness, but is not enough to determine clinical decision-making. Value-based care organizations, in contrast, are positioned to monitor real-time signals that emerge during the course of care and to act accordingly. Our results underscore the need to supplement prospective risk-based models with dynamic, system-level strategies that respond to this emerging, endogenous information, enabling providers to match resources to needs as they arise.

## Data Availability

The Limited Data Set used in this study were obtained from the Research Data Assistance Center https://resdac.org. Due to Health Insurance Portability and Accountability Act (HIPAA) regulations in the U.S., these data are not publicly available and require a Data Use Agreement.
